# Genome-wide analysis of genetic loci and candidate genes related to teat number traits in Dongliao black pigs

**DOI:** 10.3389/fgene.2025.1593395

**Published:** 2025-05-14

**Authors:** Juan Ke, Changyi Chen, Junwen Fei, Ke Luo, Yu Cheng, Huimin Yu, Chao Cheng, Yiqing Yan, Xiaoran Zhang, Shuang Liang, Hao Sun, Chunyan Bai, Boxing Sun

**Affiliations:** ^1^ College of Animal Science, Jilin University, Changchun, China; ^2^ Jilin Shuangtian Ecological Agriculture Co., LTD., Liaoyuan, China

**Keywords:** Dongliao black pig, teat number, GWAS, heritability, *RYR3*, *PIGH*

## Abstract

This study investigated the genetic basis of teat number variation in Dongliao black pigs. A total of 765 pigs were genotyped using the Porcine 50K SNP chip, and their teat numbers were recorded. Heritability estimates for total teat number (TTN) and teat pair number (TPN) were 0.091 and 0.097, respectively. Genome-wide association studies identified 74 significant SNPs for TTN and 105 for TPN. Nine candidate genes related to the teat number were identified: *CSNK1G1, PLEKHM2, CABLES1, SLC25A21, RYR3, PIGH, GUCY1A1, RAPGEF2,* and *TRPC4AP*. These findings provide insights into the genetic architecture of teat number variation in Dongliao black pigs.

## 1 Introduction

Reproduction traits such as total number of born, number of teats, and number of live born piglets are important indicators for the selection of breeding stock (Lin et al., 2024). They also represent a pivotal metric for gauging the economic impact of pig farms, in which the teat number is an important indicator of sows’ lactation capacity and lactation ability. The molecular genetic mechanisms underpinning this phenomenon play a pivotal role in the selection of superior breeding pigs (Zhuang et al., 2020), because it correlates differently with reproductive traits such as litter size and piglet survival rate (Chomwisarutkun et al., 2012). However, the teat numbers vary greatly among different pig breeds, the total teat number of the Large White pig is approximately 14, while the numbers of the Chinese local pig breed, the Erhualian pig, reaches a maximum of 25 teats. The teat number of sows is a quantitative trait, controlled by micro-effective polygenes and breed-specific. The heritability of teat number also varies significantly by breed and strain, for instance, the American Large White is 0.42 and the Danish Large White is 0.17 (Fang et al., 2022), the American Duroc heritability is 0.34, Canadian Duroc heritability is 0.19 (Rohrer and Nonneman, 2017), the heritability of Taihu pig, a local pig in China, is 0.12–0.31.

Reproductive traits such as the total number of born, number of teats, and number of live born piglets are all quantitative traits, and their phenotypes are susceptible to gene-environment interactions, and the phenotypes are usually characterized by the combined effects of multiple quantitative trait loci (QTL), so traditional breeding programs are unable to achieve more significant genetic progress. In order to explore the patterns of genetic variation in quantitative traits, the field of swine breeding has been focusing on the localization of QTLs for quantitative traits as a research priority (Korte and Farlow, 2013). To date, a large number of QTLs have been identified in different populations through research and analysis, and these QTLs have been continuously recorded in the Pig Quantitative Trait Locus Database (Pig QTLdb) (Hu et al., 2022), and there are currently more than 2,000 QTLs for teat number, accounting for about 4% of the total QTLs in the Animal Quantitative Trait Locus Database.

Genome-wide Association Study (GWAS) is a method that utilizes the genetic variation information of a population to associate with phenotypic information in order to mine candidate genes associated with target traits. This method has been widely used to analyze the genetic mechanism of economic traits in livestock and poultry (Tian et al., 2020). For example, Deng et al. identified five candidate genes, *WNT10B, AQP5, FMNL3, NUAK1,* and *CKAP4*, which are functionally related to breast development by GWAS (Deng et al., 2024). Various analytical methods based on GWAS are also becoming more sophisticated. Cai et al. showed that meta-analysis through genome-wide association studies (GWAS meta-analysis) had more accurate predictive properties compared to GWAS (Cai et al., 2024). Moscatelli et al. (2020) identified 5 SNPs in chromosome (CHR) 7 of the Large White pig. Some of the remaining studies have also screened for SNPs associated with pig teat number on many chromosomes such as CHR 7 (Zhuang et al., 2020), CHR8, and CHR13 (Uzzaman et al., 2018), respectively, and screened for candidate genes such as *VRTN, LIN52,* and *ABCD4*. These genes have been shown to be directly or indirectly associated with teat number in pigs, such as *ABCD4* is associated with mammary gland development and lactation (Guo et al., 2024; Yang et al., 2023).

The Dongliao Black Pig is a specialty breed in Liaoyuan, Jilin Province, China (Sun et al., 2024). It has excellent characteristics including adaptability, cold resistance and roughage tolerance. The breed is able to gain weight and survive the winter months with minimal facilities, demonstrating resilience to harsh conditions, effective social organization and a high farrowing survival rate in harsh housing conditions. In this study, Dongliao black pigs were used as experimental animals, and the distribution of left, right, and total teat numbers and teat pair numbers of research groups were counted, and individual genotyping was performed using the SNP chip. The heritability of teat number was estimated based on SNP chip data, and GWAS analysis between phenotypes and genotypes was performed in the whole population, combined with the relevant candidate QTLs in the pig QTL database and GWAS analysis to excavate candidate QTLs and genes significantly affecting the teat number and to functionally annotate the candidate genes, which provided theoretical references for the study of the genetic basis of the number of teats of the Dongliao black pig and the breeding improvement of breeding pigs.

## 2 Materials and methods

### 2.1 Phenotypic data

This study was conducted strictly in compliance with guidelines of experimental animals established by the Ministry of Agriculture of China, and was approved by the Laboratory Animal Welfare Ethics Committee of the Jilin University.

In the course of our experiment, we collected phenotypic data from 765 Dongliao black pigs, comprising 42 boars and 723 sows. All pigs were kept at a same farm, where all the environments and feeding materials were the same. For the purpose of further analysis, we recorded the left teat number (LTN), right teat number (RTN), total teat number (TTN), and teat pair number (TPN) of these pigs.

### 2.2 Genotypic data quality control

The study utilized the chip data of 765 Dongliao black pig genomes for SNP determination using the 50K chips. Because these microarray data were determined based on genomic data of *sus scrofa* v10.2, so the chip data were converted to align with the genomic data of *sus scrofa* v11.1 using the online tool of liftOver (https://genome.ucsc.edu/cgi-bin/hgLiftOver) (Perez et al., 2025). Subsequently, quality control is conducted in accordance with the PLINK (Chang et al., 2015). Autosomal SNP loci were retained. The samples with less than 90% SNP detection were excluded. The missing genotypes were imputed by Beagle (v5.4) (Browning et al., 2018). The loci with a minimum allele frequency (MAF) of less than 0.05 were excluded.

### 2.3 Statistical methods

HIBLUP (v1.5) (https://www.hiblup.com) (Yin et al., 2023) was used to estimate heritability and breeding values for LTN, RTN, TTN and TPN based on the above chip data and phenotypic values. Each trait was analyzed using a single trait animal model. The model equation is y=Xb+Za+e , where y is the vector of phenotypic values for the trait; b is the vector of fixed effects (mean term and sex); a is the vector of additive genetic effects (breeding values); e is the vector of residual effects; X and Z are the correlation matrices corresponding to b and a , respectively. Heritability was estimated as $h^{2}=\frac{\sigma_{a}^{2}}{\sigma_{a}^{2}+\sigma_{e}^{2}}$ , where $\sigma_{a}^{2}$ is the individual additive genetic variance and $\sigma_{e}^{2}$ is the residual variance.

GWAS was conducted using the Bayesian-information and Linkage-disequilibrium Iteratively Nested Keyway (BLINK) (Huang et al., 2019) and Fixed and random model Circulating Probability Unification (FarmCPU) (Liu et al., 2016) models in GAPIT (v3) (Wang and Zhang, 2021) based on the estimated breeding value data. Principal components (PC) were subjected to analysis using the GAPIT. The initial three PC were used as covariates. The core code of GAPIT used for the analysis is as follows:

“GAPIT(Y = y, GD = X, GM = map, model = c (“Blink”, “FarmCPU”), PCA. total = 3, SNP.MAF = 0.05)”.

Significant SNPs were annotated through the Pig QTLdb (https://www.animalgenome.org/cgi-bin/QTLdb/SS/index) (Hu et al., 2022) and the online tool VEP (https://www.ensembl.org/info/docs/tools/vep/index.html). The function of genes were confirmed in the Ensembl database (https://www.ensembl.org) (Harrison et al., 2024). Gene Ontology (GO) and Kyoto Encyclopedia of Genes and Genomes (KEGG) pathway enrichment analysis were conducted using the DAVID online tool (https://david.ncifcrf.gov/tools.jsp). We checked and screened the genes through PigBiobank (Zeng et al., 2024) and iswine (Fu et al., 2020) databases. Candidate genes prioritization was primarily based on the *p*-values from the GWAS results, and then considered the pig QTLdb and gene functions.

## 3 Results

### 3.1 Results of statistical data and genetic parameters

Phenotypic data were collected from 765 pigs and statistically analyzed for number of left teats, right teats, total teats, and teat pair numbers (Table 1). The mean (mean ± standard deviation) of LTN, RTN, TTN, and TPN were 6.36 ± 0.61, 6.47 ± 0.62, 12.83 ± 1.19, and 6.42 ± 0.59, respectively, and their coefficients of variation (C.V.) were in the range of 9%–10%. The heritability was calculated based on the 35,096 SNPs obtained after quality control, and the heritability estimates of the four traits were 0.083, 0.090, 0.091, and 0.097. Since the heritability of the teat pair numbers was higher than that of the other three traits, the GWAS analysis was conducted on TPN, while TTN was used as a reference.

**TABLE 1:** Variance components and heritability of the traits.

Trait	Mean (±SD)	Min	Max	C.V. (%)	h^2^(SE)	*p*
LTN	6.36 ± 0.61	5	8	9.66	0.083 ± 0.040	0.032
RTN	6.47 ± 0.62	5	8	9.55	0.090 ± 0.040	0.024
TTN	12.83 ± 1.19	10	16	9.27	0.091 ± 0.040	0.023
TPN	6.42 ± 0.59	5	8	9.26	0.097 ± 0.041	0.017

LTN, left teat number; RTN, right teat number; TTN, total teat number; TPN, teat pair number; C.V.: coefficient of variation; $h^{2}$ (SE): Heritability (Standard error).

### 3.2 GWAS analyze

**FIGURE 1:**
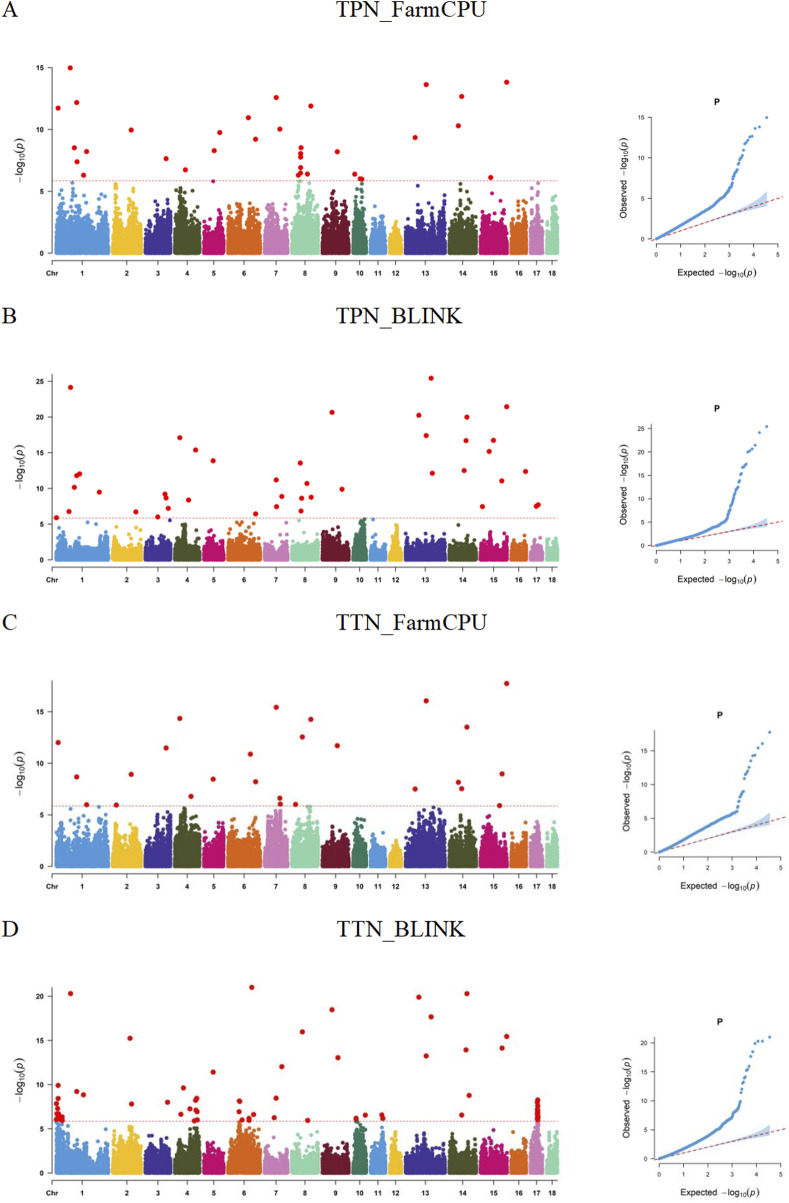
Manhattan and QQ plots for the teat pair number (TPN) and the total teat number (TTN) traits based on the BLINK and FarmCPU models. The red line represents the genome-wide significant threshold and the value is 5.846.

The estimated breeding values for TPN and TTN were used for GWAS analysis. PCA plot are shown in the Supplementary Figure S1. The genome-level threshold of significance was 5.846 ( $p={-\log}_{10}\left(\frac{0.05}{35096}\right)$ ). Many significant SNPs were detected in both traits. The GWAS results using the BLINK model identified 42 and 87 SNPs significantly associated with TPN and TTN, respectively, and GWAS results using the FarmCPU model identified 38 and 26 SNPs significantly associated with TPN and TTN, respectively (Supplementary Figure S1). The Manhattan and Q-Q plots of the GWAS results are shown in Figure 1. These SNPs were distributed on all chromosomes. All the SNPs are listed in Supplementary Tables S1, S2.

### 3.3 Annotation and enrichment analysis

We annotated all significant loci from the GWAS results using bedtools software in combination with QTL databases and Ensembl annotations. Focusing on QTLs associated with reproductive traits, we identified a total of six significant SNP loci (Supplementary Table S3), four of which were specifically linked to the nipple count trait. Notably, the loci on chromosomes 14 and 17 were each mapped to specific genes: *LIMK2* and *TRPC4AP*, respectively. Through Ensembl annotation, these SNPs were mapped to a total of 84 genes (all possessing official gene IDs), with 74 assigned specific gene names (Supplementary Material S4). Notably, this set included *LIMK2* and *TRPC4AP*. A comprehensive evaluation was conducted to investigate the relationship between specific genes and pig teat number trait, this involved an extensive review of relevant databases, primarily PigBiobank and iswine alongside a detailed analysis of associated literature, aimed at identifying loci and candidate genes that have been either confirmed or proposed to be associated with this trait. The findings from this investigation are summarized in Table 2. The results of these genes annotation were analyzed by enrichment in the GO database and the KEGG database by DAVID. The KEGG pathway of Oxytocin signaling pathway (ssc04921), the *GUCY1A1* and *RYR3* genes have been implicated in this pathway. Based on this evidence, we propose that these two genes are likely the primary contributors to the variation in pig teat number.

**TABLE 2:** Candidate genes and related information.

CHR	POS	*p*_value	Traits	Gene	Related study
1	1.08E+08	2.11E-09	F.TT	*CSNK1G1*	Ding et al. (2009)
6	74903107	9.97E-07	B.TT	*PLEKHM2*	Zhang et al. (2007)
6	1.08E+08	1.13E-11	F.TP	*CABLES1*	Tortereau et al. (2010)
7	63140312	3.62E-08	B.TP	*SLC25A21*	Wada et al. (2000)
7	80869930	2.44E-07	F.TT	*RYR3*	Cassady et al. (2001)
7	91308348	9.51E-13	B.TT	*PIGH*	Ding et al. (2009)
8	44518028	2.82E-14	B.TP	*GUCY1A1*	Sato et al. (2006)
8	48263563	1.49E-07	B.TP	*RAPGEF2*	Verardo et al. (2016)
17	38387643	1.01E-07	B.TT	*TRPC4AP*	Verardo et al. (2015)

F means FarmCPU model of GWAS; B means BLINK model of GWAS; TT means the trait of total teat number; TP means the trait of teat pair number.

## 4 Discussion

Sows’ milk constitutes a source of nutrition for newborn piglets. The capacity of the sow to produce milk exerts a profound influence on the growth and health status of the piglets, with these effects persisting even after weaning. The lactation capacity of sows can be enhanced by the number of teats they possess, a trait that is also heritable. The mean values of LTN, RTN, and TTN in this study were 6.36, 6.47, and 12.83, respectively, and this number is lower than that of some Chinese local pig breeds (Erhualian pig (Tang et al., 2017), Qingping pig (Liu et al., 2022)) and some commercial pig breeds in other countries (Large White, Landrace (van Son et al., 2019)). In this study, the heritability of the teat pair number of Dongliao Black pigs was 0.097 and the heritability of the total teat number was 0.090, which were significantly lower than the rest of the breeds that have been reported (Fang et al., 2022; Rohrer and Nonneman, 2017; van Son et al., 2019), suggesting that the heritability of the trait is low. However, the current study showed that the number of teats in pigs is less influenced by the environment and more influenced by genetic factors (Li et al., 2021). Therefore, we believe that it is more difficult for Dongliao Black pigs to increase the number of teats and can be genetically stabilized by within-population selection, and whether we should consider breeding by crossbreeding with populations with high heritability for teat number.

In this study, we screened 74 and 105 SNPs associated with TPN and TTN, respectively (Supplementary Tables S1, S2), and these SNPs screened were basically distributed on all chromosomes, which is consistent with the previous results on other reproductive traits in Dongliao black pigs (Sun et al., 2024), this suggests that the genes affecting reproductive traits including teat number may be the same, and that all of these reproductive traits are under the control of multiple genes.

Genetically annotating these SNPs, we identified some key genes, *CSNK1G1, PLEKHM2, CABLES1, SLC25A21, RYR3, PIGH, GUCY1A1, RAPGEF2* and *TRPC4AP*. And *TRPC4AP* were reported to be associated with porcine teat number in the QTL database. *PIGH* was found to be associated with the metabolism of milk fat in dairy cows (Feng et al., 2024), and Zhou et al. (Jiawei et al., 2022) also identified *PIGH* as one of the candidate genes affecting the number of teats in Canadian line Large White pigs in their study as well. *RYR3* is ryanodine receptor 3, one of the cellular calcium ion transport proteins, and is associated with meat quality (Wu et al., 2020), so we considered that it might be associated with the number of teats in pigs. Based on the aforementioned analysis, it can be conclusively demonstrated that these genes play a crucial regulatory role in determining teat number in Dongliao black pigs. Through GO and KEGG enrichment analyses, we identified that both *GUCY1A1* and *RYR3* are significantly involved in the oxytocin-mediated signaling pathway (ssc04921). Based on this evidence, we propose that *GUCY1A1* and *RYR3* represent the key regulatory genes influencing teat numbers. Our future research efforts will focus on conducting functional validation studies to confirm their specific roles in regulating teat number.

## 5 Conclusion

In our study, the heritability of the teat number trait in Dongliao black pigs were 0.091 (TTN) and 0.097 (TPN). Through GWAS, we identified a total of 74 significant SNPs associated with TPN and 105 significant SNPs associated with TTN at the genome-wide level. Furthermore, we identified nine candidate genes associated with teat number, namely *CSNK1G1, PLEKHM2, CABLES1, SLC25A21, RYR3, PIGH, GUCY1A1, RAPGEF2* and *TRPC4AP*. Notably, 2 genes (*GUCY1A1* and *RYR3*) were enriched in the Oxytocin signaling pathway. These findings provide valuable insights into the genetic mechanisms underlying teat number variation in Dongliao black pigs and offer a foundation for further investigations into optimizing reproductive performance in pig breeds.

## Data Availability

The datasets presented in this study can be found in online repositories. The names of the repository/repositories and accession number(s) can be found in the article/Supplementary Material.
