# Supplementary material for: Genome-wide analysis of genetic loci and candidate genes related to teat number traits in Dongliao black pigs
Source: Front Genet. 2025 May 14;16:1593395. doi: 10.3389/fgene.2025.1593395 (PMC12116542; doi:10.3389/fgene.2025.1593395)
Supplement: Supplementary file 1 [file DataSheet1.zip › Supplementary_Material.docx]

Supplementary Material

# Supplementary Figures
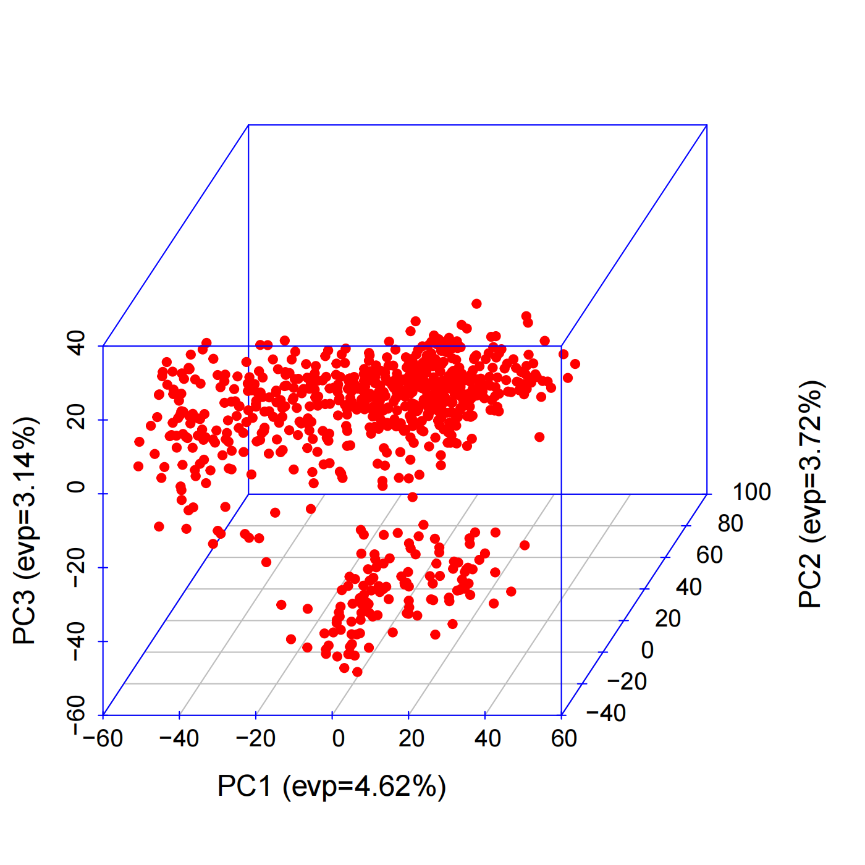


## Supplementary Figure 1. PCA plot of our samples.


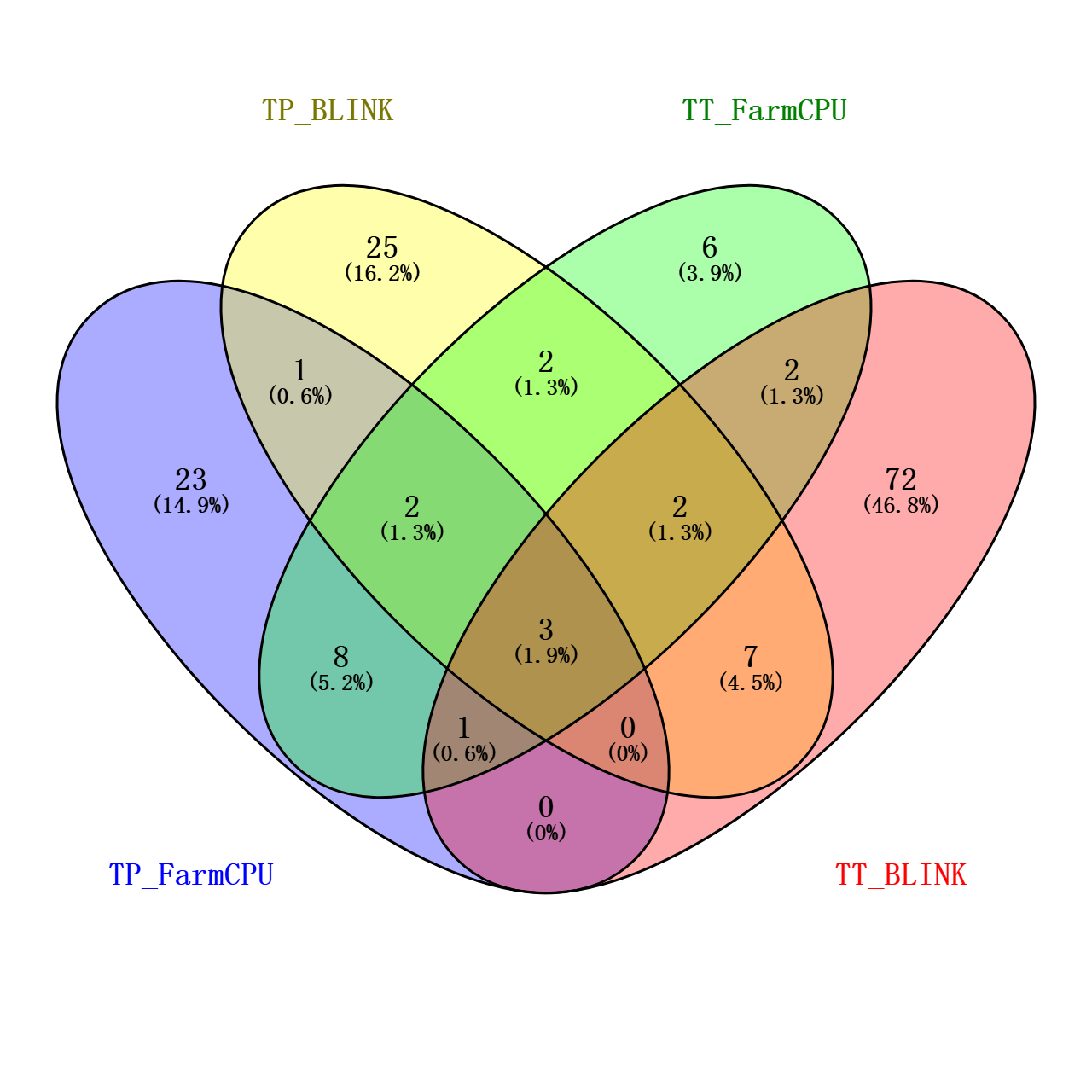


## Supplementary Figure 2. Veen plot of significant SNPs of evety traits. TT means Total teat number, TP means teat pair number, FarmCPU and BLINK were two model of Genome-wide association studies (GWAS).
